# Normothermic *Ex Vivo* Machine Perfusion of Discarded Human Pancreas Allografts: A Feasibility Study

**DOI:** 10.3389/ti.2023.10936

**Published:** 2023-05-11

**Authors:** Catherine Parmentier, Samrat Ray, Laura I. Mazilescu, Masataka Kawamura, Yuki Noguchi, Emmanuel Nogueira, Sujani Ganesh, Bhranavi Arulratnam, Sangeetha N. Kalimuthu, Markus Selzner, Trevor W. Reichman

**Affiliations:** ^1^ Toronto General Hospital, Toronto, ON, Canada; ^2^ University Health Network (UHN), Toronto, ON, Canada; ^3^ Essen University Hospital, Essen, North Rhine-Westphalia, Germany

**Keywords:** pancreas transplantation, perfusion, normothermic machine perfusion, diabetes, human pancreas allografts

## Abstract

Pancreas transplantation is the only curative treatment for patients with complicated diabetes, and organ shortage is a common and increasing problem. Strategies to expand the donor pool are needed, and normothermic *ex vivo* perfusion of the pancreas has the potential to test and repair grafts before implantation. Between January 2021 and April 2022, six human pancreases, declined for transplantation or islet isolation, were perfused using a previously established method by our group. All 6 cases were successfully perfused for 4 h, with minimal edema. The mean age of the donors was 44.16 ± 13.8 years. Five grafts were obtained from neurological death donors, and one was obtained from a donation after cardiac death. The mean glucose and lactate levels decreased throughout perfusion and insulin levels increased. All 6 grafts were metabolically active during perfusion and histopathology showed minimal tissue injury and no edema. Human normothermic *ex vivo* perfusion of the pancreas is feasible and safe and has the potential to expand the donor pool. Future studies will focus on tests and biomarkers for the assessment of grafts.

## Graphical Abstract

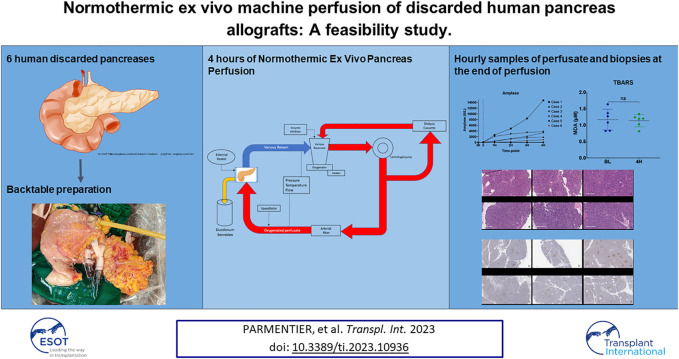

## Introduction

Despite advances in insulin pump technologies and therapies, pancreas transplantation (PTx) is still the only curative treatment for patients with diabetes [1]. Historically, PTx either simultaneous (SPK) or pancreas after kidney (PAK) has been performed in patients with type 1 diabetes mellitus and concomitant kidney disease requiring a renal transplant. In select cases, pancreas transplant alone (PTA) has been performed in patients with life-threatening hypoglycemic unawareness. More recently the indications for PTx have been expanded and performed in patients with a diagnosis of type 2 diabetes mellitus with comparable results [2–4]. Broadening of the acceptance criteria for recipients has led to an increasing need for suitable pancreas grafts. However, the pool of suitable pancreas donors has remained largely stagnant [5].

There is a significant benefit to recipients of pancreas allografts demonstrating improvement in both quality of life and life expectancy [6, 7]. Although PTx does not reverse complications associated with diabetes, it has been shown to decrease the predicted cardiovascular risk by more than two-thirds at 5 and 10 years [1, 8].

Even with an increasing need for pancreas grafts, donor selection continues to be very restrictive, and the conversion rate from donation to transplant remains low [9]. In addition, the pancreas continues to be the most discarded organ. In Canada, in 2019, out of a total of 820 deceased donors, only 68 pancreases were transplanted [10]. In the UK, only 1/3 of accepted pancreases are transplanted [9, 11]. Not surprisingly, the number of PTx performed is considerably lower as compared to kidney, liver, heart, and lung [9, 12]. Strategies that will allow for the assessment and repair of pancreas allografts have the potential to reverse this trend in pancreas donation. Normothermic *ex vivo* machine perfusion (NEVPP) has been successfully used for the preservation of liver [13, 14], kidney [15, 16], heart [17], and lung allografts [18] but has only scarcely been studied for the pancreas.

Earlier studies of NEVPP have been limited as grafts develop severe edema and tissue injury [19, 20]. However, our previous work in a porcine model demonstrated that edema can be mitigated and that these grafts can be successfully transplanted after 3 h of perfusion [21]. The purpose of this study was to prove the feasibility and safety of this method in human allografts.

## Material and Methods

Between January 2021 and April 2022, we received 7 human pancreas allografts recovered from multiorgan donors in Ontario, Canada. These grafts were declined for pancreas transplantation and islet cell isolation but donated for research purposes. The study was approved by the medical ethical committee of the Toronto General Hospital (Approval number: 20-5733). The allografts were retrieved by the multiorgan procurement team at Toronto General Hospital. All allografts were flushed and stored in University of Wisconsin (UW) preservation solution (Bridge to Life, London, United Kingdom). One of the grafts had to be discarded because of technical issues (heating pump failure) during the perfusion that did not allow for appropriate data collection.

### Allograft Preparation

**FIGURE 1:**
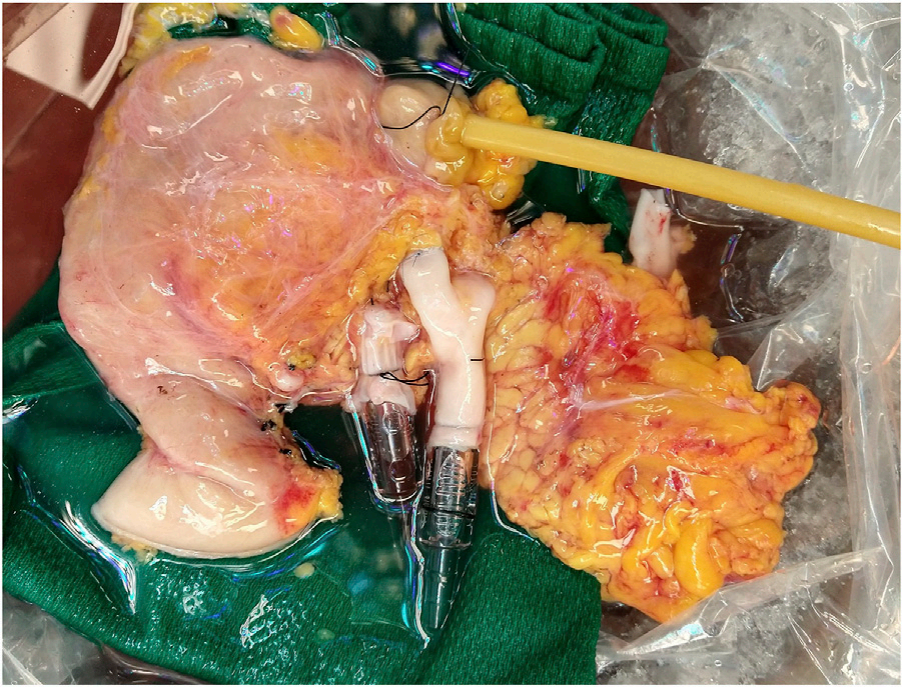
Pancreas allograft after backtable.

Recovered pancreas allografts were prepared for NEVPP utilizing a backtable preparation typical for human pancreas implantation. Briefly, the organ was inspected for any significant damage that would affect the perfusion. The spleen was removed by ligating the splenic artery and vein close to the hilum of the spleen. Iliac vessels were recovered from the donor and any small branches were suture ligated. Arterial reconstruction was performed using the donor iliac artery as a “Y graft.” The external iliac artery and internal iliac artery were anastomosed to the splenic artery and the superior mesenteric artery with a 6-0 polypropylene suture, respectively. The common iliac artery was then used for cannulation. Similarly, an iliac vein was used as an extension graft by anastomosing the iliac vein to the graft portal vein in an end-to-end fashion using 6-0 polypropylene suture [22]. The artery and vein were cannulated with 1/4″ x 3/8″ reducers. The bowel was shortened if necessary and a Malecot catheter (Bard, 22 Fr, Covington, GA, USA) was inserted into the distal end to collect duodenal and pancreatic exocrine output during perfusion (Figure 1). The pancreas was weighed after completion of the back table and then flushed with 200 mL of 5% albumin before initiating NEVPP.

### Normothermic *Ex Vivo* Perfusion

**FIGURE 2:**
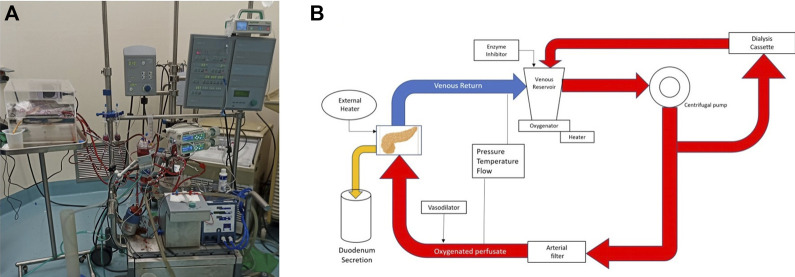
Normothermic Machine. **(A)** Photography. **(B)** Schematic of the circuit.

**FIGURE 3:**
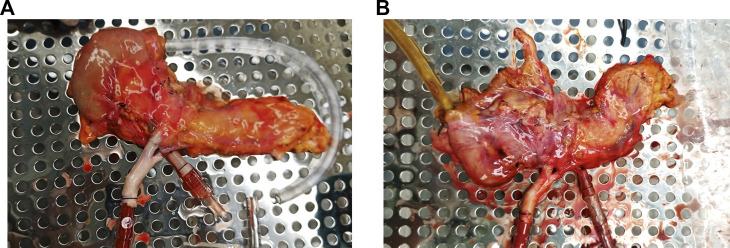
Pancreas allograft. **(A)** at the beginning of perfusion. **(B)** At the end of perfusion.

Pancreas allografts were perfused for 4 h using the method described previously by our group [21, 23]. Briefly, a neonatal cardiopulmonary bypass system was used and fitted with a custom-made circuit (Sorin Group Canada Inc., Markham, Canada). In this system, the perfusate travels from the venous reservoir with the help of a centrifugal pump into an oxygenator. Following oxygenation, the circuit divides into two circuits with a part of the perfusate circulating through a dialysis filter and then back to the reservoir. The second circuit passes blood through an arterial bubble filter and then into the pancreas graft. The venous outflow goes back into the venous reservoir [21, 23] (Figure 2). The first 4 grafts were perfused with an O2/CO2 concentration of 95/5% and the last 2 grafts were perfused with a concentration of 91/9%. Figure 3 shows a graft at the beginning and at the end of the perfusion. The perfusate’s composition is shown in Table 1. Dialysate is infused at a rate of 1 L per hour and prepared before every experiment. The dialysate consisted of 22 mL of 45X concentrated hemodialysis solution (Baxter Corporation), 27 mL of 8.4% sodium bicarbonate, 3 mL of 8.4% potassium bicarbonate, 275 mg of sodium pyruvate, and 1.5 g of NaCl. The volume was then brought up to a liter with double reverse osmosis water.

**TABLE 1:** Perfusate composition.

Ingredient	
Steen Solution	215 mL
Packed red blood cells	400 mL
Sodium bicarbonate (8.4%)	10 mL
Heparin (10000 IU/10 mL)	1.3 mL
Aprotinin	15 mg *
Continuous infusion:	
Epoprostenol – 0.5 mg dissolved in 250 mL of ringer’s lactate and infused at 8 mL/h.	
*Aprotinin – 30 mg dissolved in 60 mL of ringer’s lactate. 30 mL (15 mg) go are directly poured into the reservoir and the rest is infused at 10 mL/h.	

A 4-hour perfusion time was decided upon since we believe that at least 2 h of perfusion are needed to perform any intervention on the graft. Doubling that period seemed to be a reasonable starting point for our first feasibility study with human grafts.

During the perfusion, arterial pressure and flow were measured and recorded every hour. Blood gas analysis from the perfusate was used to record acid-base and electrolyte balance and samples were taken every hour for storage. Duodenal output was measured every hour and recorded if present.

### Histology

A core biopsy (Bard, Monopty disposable core biopsy instrument, 14g × 16 cm, Georgia, USA) was taken from the tail before the start of the perfusion, at 1-hour of perfusion. At the end of the perfusion 4 wedge biopsies were taken from the head, body, tail, and duodenum. These biopsies were fixed in formalin, snap frozen, and stored in RNA later (Stabilization Solution, Invitrogen, Thermo Fisher Scientific.

All the formalin samples (10% neutral buffered formalin) were stored for 48 h and then transferred to 70% alcohol. They were then all sent for paraffin block embedding and hematoxylin and eosin (H&E) staining. A semiquantitative scale, developed by our pathologist, was used to score fat and parenchyma necrosis (0 - no changes, 1 - mild changes, 2 - moderate changes, 3 - severe changes) [21].

For the assessment of islet cells, additional insulin staining was performed and reported as number if islet cells at a ×4 magnification. For the assessment of apoptosis, a TUNEL assay was performed on the end of perfusion samples and reported as negative, <30%, 30%–60% or >60%. For the assessment of vascularity of the grafts, a CD31 staining was performed. Interstitial edema was assessed on histopathology and classified as none, mild, moderate, or severe. All the histopathological analysis was performed by a GI/pancreas pathologist.

### Oxidative Stress

Samples of the perfusate were stored at −80°C. These samples were thawed and used to measure thiobarbituric acid reactive substances (TBARS) using a commercial assay kit (OxiSelect TBARS Assay Kit, Cell Biolabs, Inc.)

### Data Analysis and Statistics

Continuous data are represented as mean and standard deviation and plotted versus time for each case. GraphPad Prism Software 9 was used for analysis and graphs.

For each case, the following variables were collected: age, cause of death (COD), type of donor (NDD or DCD), gender, height, BMI, cold ischemia time (CIT), blood type, and reason for discard.

## Results

### Donor Characteristics

Characteristics of the donors and cold ischemia times are shown in Table 2. Half of the donors were male. Of the 6 included cases only one graft came from a DCD donor, with a warm ischemia of 17 min The mean age was 44.16 ± 13.79 years. The cause of death was anoxia in 2 donors, cardiac arrest in 2 donors, CVA/stroke in 1 donor and, head trauma in 1 donor. The mean cold ischemia time was 372.50 ± 137.69 min with a range of 173–547 min. The mean height was 172.50 ± 14.42 cm. The mean weight was 78.46 ± 25.70 kg and, and the mean BMI was 25.71 ± 5.81. The reason for discard was fatty infiltration in 2 grafts, older donor in 2 cases and high BMI in one case. Four out of the six donors presented a cardiac arrest event that required CPR and five out of six required vasopressors.

**TABLE 2:** Donor characteristics.

	Case 1	Case 2	Case 3	Case 4	Case 5	Case 6
Age (mean ± SD) (Range)	44.16 ± 13.79 (26–62)					
Height (cm) (mean ± SD) (Range)	172.50 ± 14.42 (151–183)					
Weight (kg) (mean ± SD) (Range)	78.46 ± 25.70 (38.8–114.8)					
BMI (kg/m^2^) (mean ± SD) (Range)	25.71 ± 5.81 (17–34.3)					
CIT (minutes) (mean ± SD) (Range)	372.50 ± 137.69 (173–547)					
Gender	Male	Male	Male	Female	Female	Female
Type of donor	NDD	NDD	NDD	NDD	DCD	NDD
Cause of death	CVA/Stroke	Anoxia	Anoxia	Cardiac arrest	Cardiac arrest	Head Trauma
Blood type	O positive	A positive	B positive	A positive	A positive	B positive
WIT (minutes)	N/A	N/A	N/A	N/A	17	N/A
Reason for Discard	BMI	Fatty infiltration of the graft	Fatty infiltration of the graft	Fatty infiltration of the graft	Age	Age

### Graft characteristics

**FIGURE 4:**
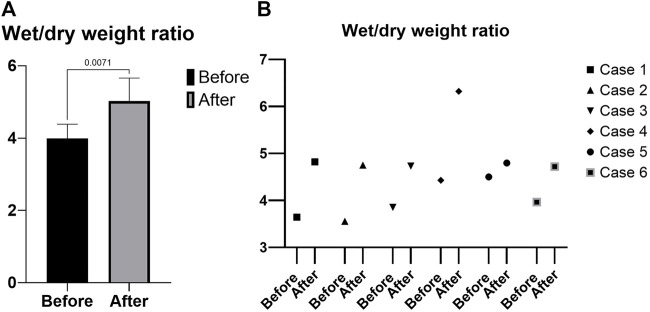
Wet/dry weight ratio. **(A)** Mean. **(B)** Individual values.

All the grafts (pancreas and duodenum) perfused evenly during the 4 h of perfusion, without any macroscopic evidence of poor circulation. The mean wet/dry weight ratio was 3.99 ± 0.39 before perfusion and 5.02 ± 0.63 after perfusion (*p* = 0.007) (Figure 4A) and a change in ratio that ranges from 6% to 42%. Individual values are shown in Figure 4B.

### Laboratory Results

**FIGURE 5:**
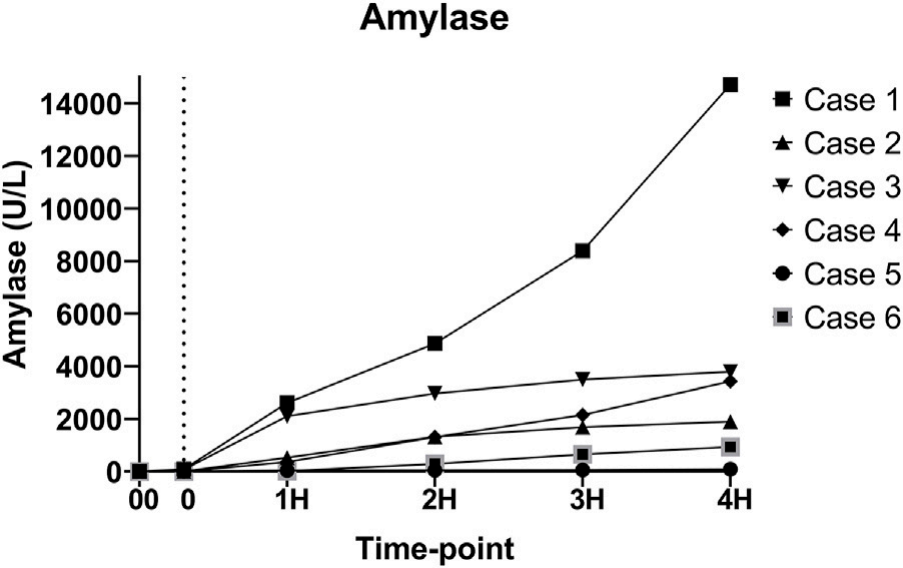
Amylase levels during perfusion. (00 - before pancreas on pump, 0 – right after pancreas on pump, 1H - 1 h, 2H −2 h, 3H – 3 h, 4H – 4 h).

**FIGURE 6:**
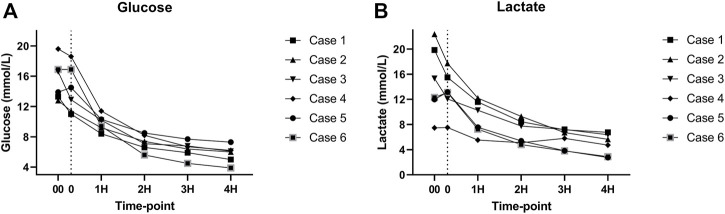
**(A)** Glucose levels during perfusion. **(B)** Lactate levels during perfusion. (00 - before pancreas on pump, 0 – right after pancreas on pump, 1H - 1 h, 2H −2 h, 3H – 3 h, 4H – 4 h).

**FIGURE 7:**
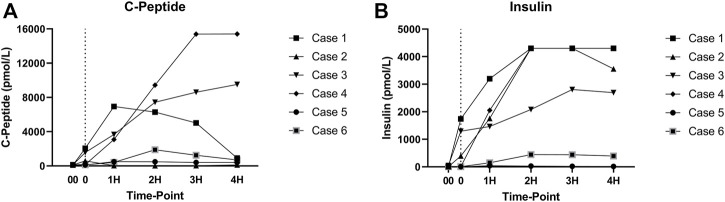
**(A)** C-peptide levels during perfusion. **(B)** Insulin levels during perfusion. (00 - before pancreas on pump, 0 – right after pancreas on pump, 1H - 1 h, 2H −2 h, 3H – 3 h, 4H – 4 h).

As expected, amylase levels increased during the 4 h of perfusion (median: 796.5U/L, IQR: 2430.75) (Figure 5). No significant difference was noted between amylase of the CO2 5% group vs. CO2 9% group. Glucose and lactate levels decreased during the 4 h of perfusion (median: 7.55 mmol/L, IQR: 4.025 mmol and median: 7.18 mmol/L, IQR: 4.59 mmol/L, respectively) (Figure 6). C-peptide levels (median: 1,084.5 pmol/L, IQR: 5559.75 pmol/L) during perfusion were more variable between cases (Figure 7A), and insulin levels increased in all the cases during the perfusion, except for case 5 (Figure 7B).

**FIGURE 8:**
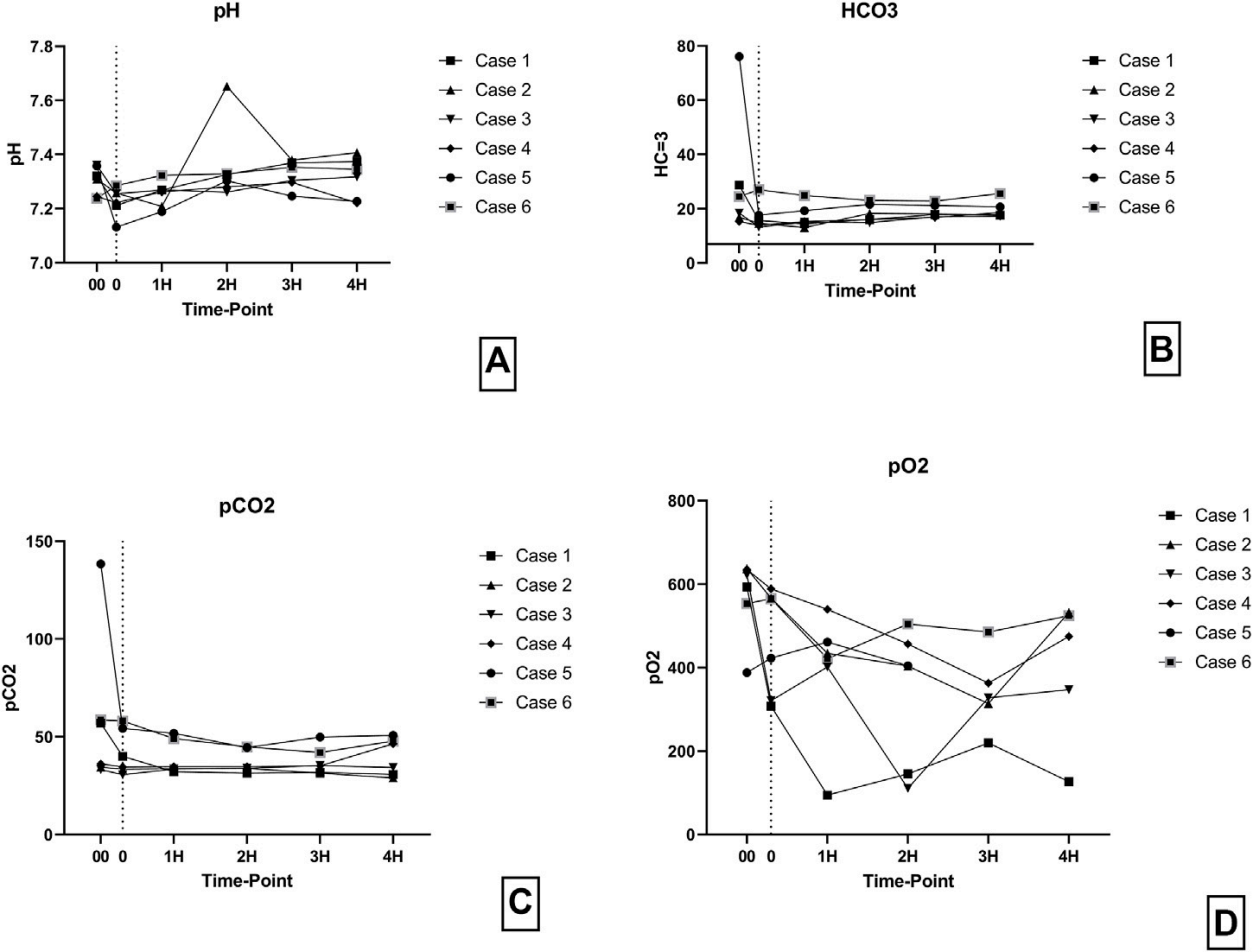
**(A)** pH levels during perfusion. **(B)** HCO3 levels during perfusion. **(C)** pCO2 levels during perfusion. **(D)** pO2 levels during perfusion. (00 - before pancreas on pump, 0 – right after pancreas on pump, 1H - 1 h, 2H −2 h, 3H – 3 h, 4H – 4 h).

Levels of pH, HCO3 and, pCO2 were consistent throughout the perfusion (Figures 8A–C). However, pO2 levels were more variable during the perfusion but were always above 100 mmHg (Figure 8D).

### Perfusion Characteristics

**FIGURE 9:**
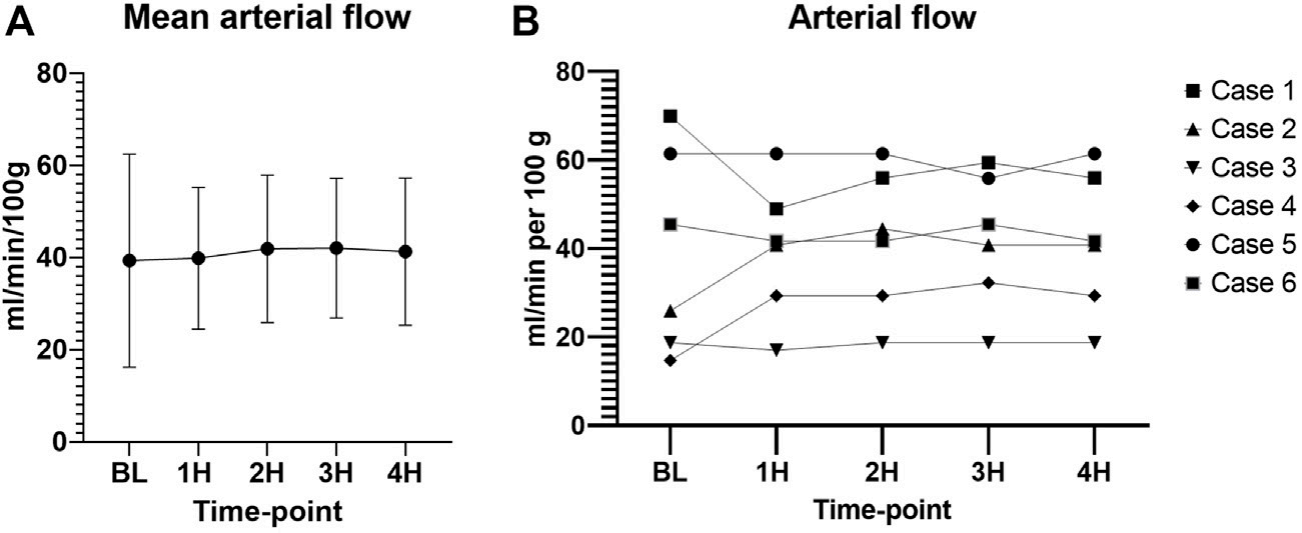
Arterial flow during perfusion. **(A)** Mean values with standard deviation. **(B)** Individual values. (00 - before pancreas on pump, BL – right after pancreas on pump, 1H - 1 h, 2H −2 h, 3H – 3 h, 4H – 4 h).

The arterial flow was stable throughout the 4 h of perfusion with a mean of 40.9 ± 16.19 mL/min/100 g (Figure 9).

**FIGURE 10:**
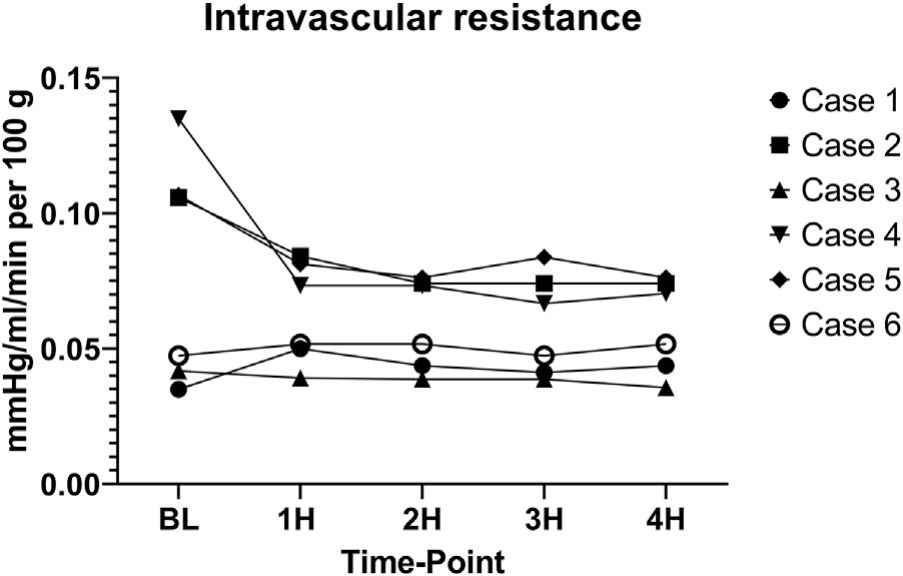
Intravascular resistance. (BL – baseline, 1H - 1 h, 2H −2 h, 3H – 3 h, 4H – 4 h).

Intravascular resistance was slightly higher for cases 2 (0.082 ± 0.013 mmHg/ml/min per 100 g), 4 (0.083 ± 0.028 mmHg/ml/min per 100 g), and 5 (0.084 ± 0.002 mmHg/ml/min per 100 g) as compared to cases 1 (0.042 ± 0.005 mmHg/ml/min per 100 g), 3 (0.038 ± 0.002 mmHg/ml/min per 100 g), and 6 (0.049 ± 0.002 mmHg/ml/min per 100 g) with a significant difference between means (*p* < 0.001) (Figure 10). CO2 5% group seemed to have a lower mean intravascular resistance than the CO2 9% group, but the difference was not statistically significant (*p* = 0.31).

### Histopathology

**FIGURE 11:**
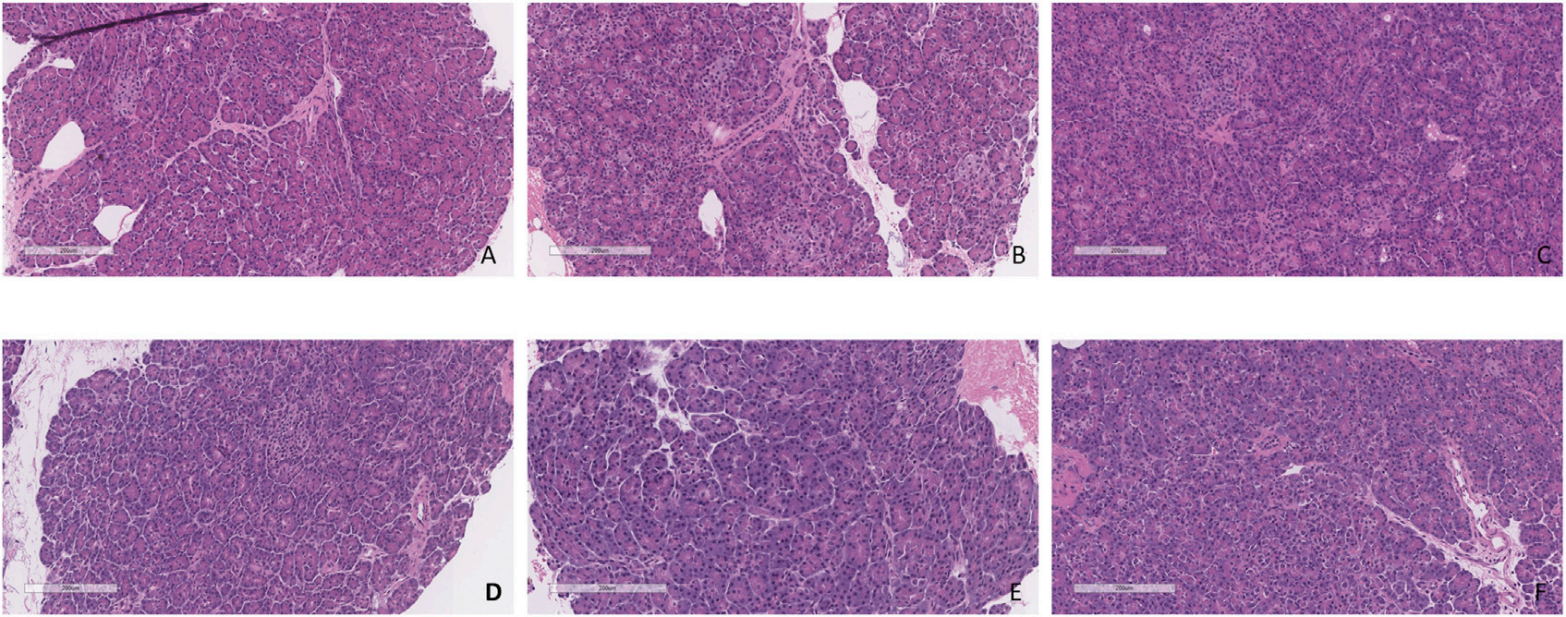
H&E staining of pancreas tail biopsies. **(A)** CO2 5% before perfusion. **(B)** CO2 5% at 1 h of perfusion. **(C)** CO2 5% at the end of 4 h of perfusion. **(D)** CO2 9% before perfusion. **(E)** CO2 9% at 1 h of perfusion. **(F)** CO2 9% at the end of 4 h of perfusion. Bar = 200 µm.

**FIGURE 12:**
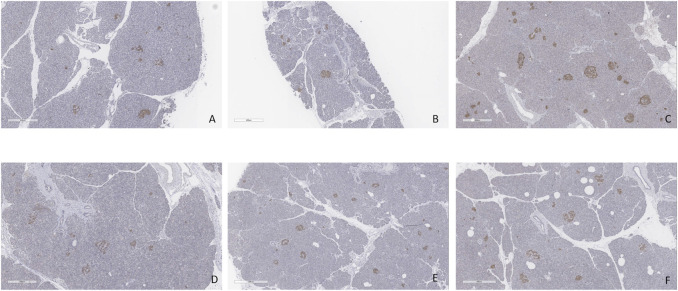
Insulin staining. **(A)** Case 1. **(B)** Case 2. **(C)** Case 3. **(D)** Case 4. **(E)** Case 5. **(F)** Case 6. All biopsies taken at the end of perfusion (4 h). Bar = 500 µm.

**FIGURE 13:**
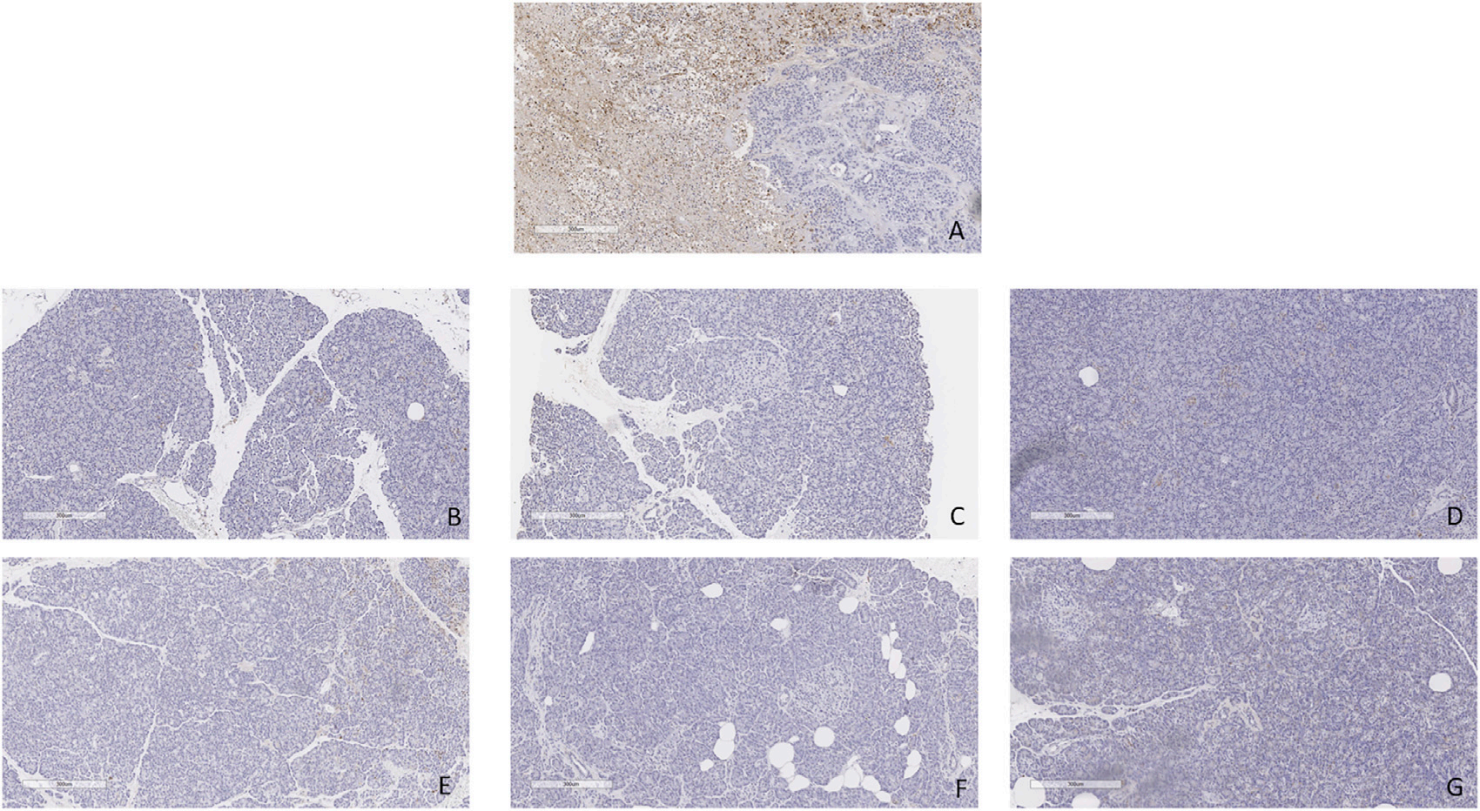
TUNEL staining. **(A)** Positive and negative control. **(B)** Case 1. **(C)** Case 2. **(D)** Case 3. **(E)** Case 4. **(F)** Case 5. **(G)** Case 6. All biopsies taken at the end of perfusion (4 h). Bar = 300 µm.

**FIGURE 14:**
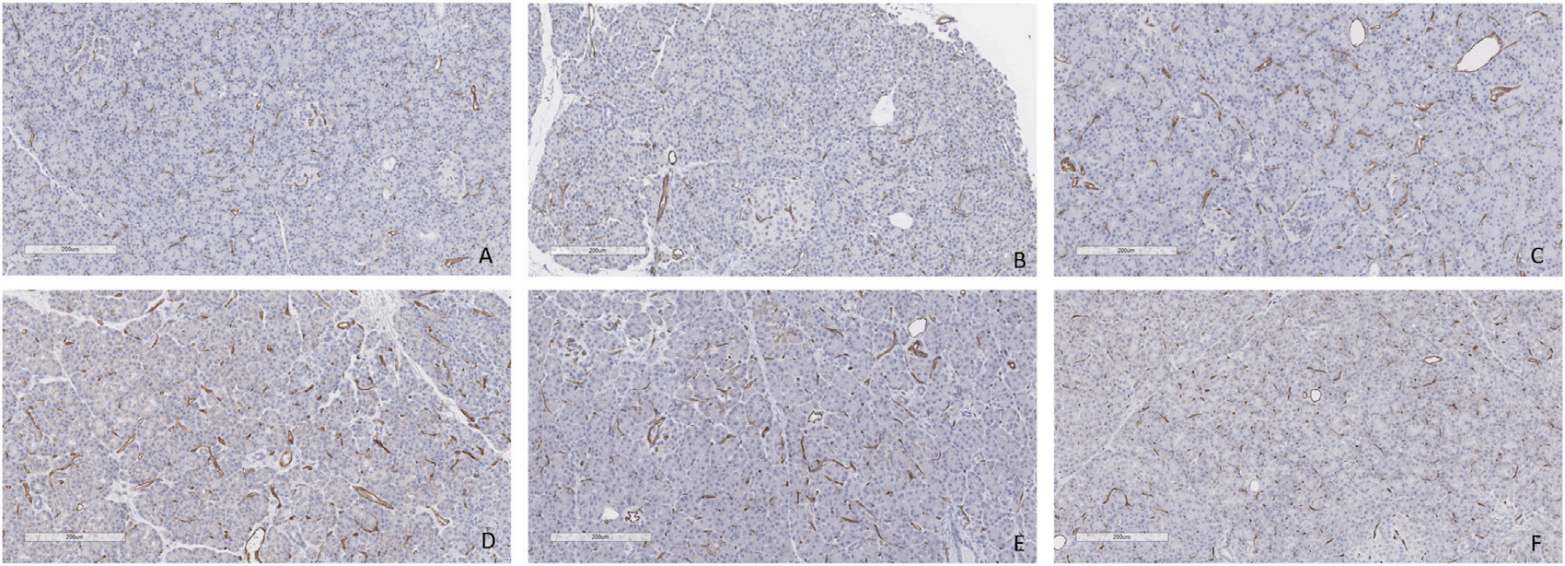
CD31 staining at the end of the perfusion. **(A)** Case 1. **(B)** Case 2. **(C)** Case 3. **(D)** Case 4. **(E)** Case 5. **(F)** Case 6. All biopsies taken at the end of perfusion (4 h). Bar = 200 µm.

Minimal tissue injury was noted in both the CO2 5% and CO2 9% groups and grafts from both groups were morphologically normal (Figure 11). Overall, the parenchyma was largely intact, with very mild focal necrosis, normal ducts, and mild hemorrhage/congestion. The duodenum showed mild to moderate erosive changes and mild autolysis. Islet cells were present in all the cases (Figure 12). No edema was observed in any of the grafts and TUNEL assay was negative for all the cases except for case 1 which presented less than 30% (approximately 5%) (Figure 13). All grafts were vascularized at the end of the perfusion as seen in the pancreatic tissue stained with CD31, with no evidence of thrombosis (Figure 14).

### Oxidative Stress

**FIGURE 15:**
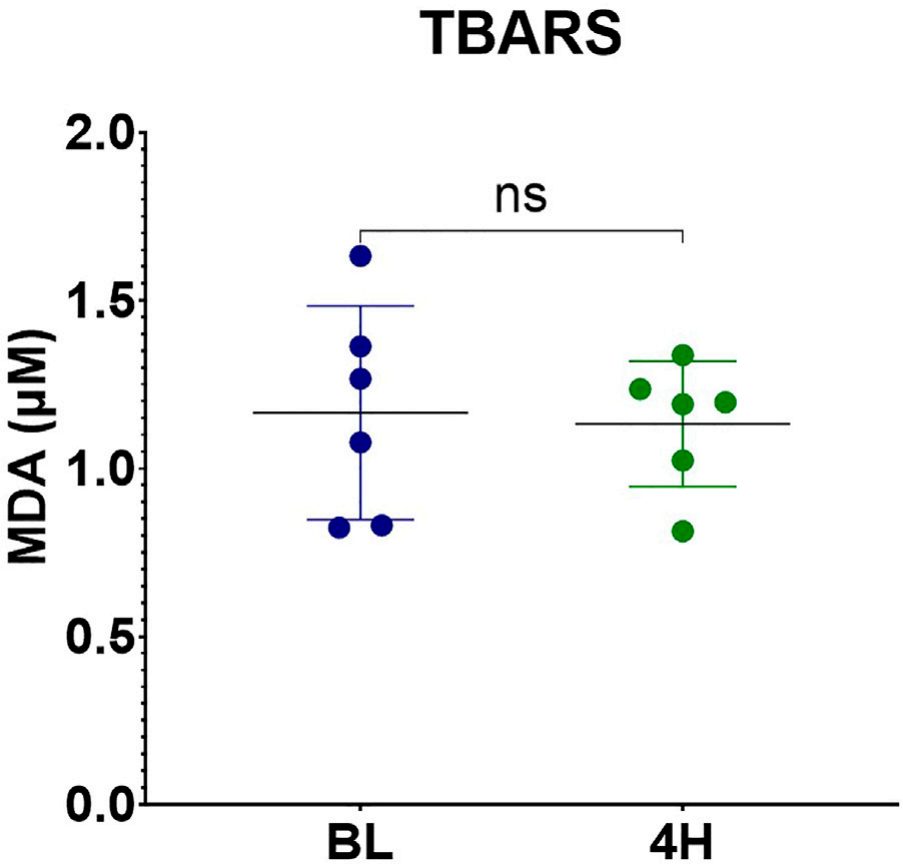
MDA levels measured by TBARS assay.

TBARS were measured from the perfusate at baseline and at the end of the perfusion. No significant difference was noted between samples at baseline and at the end of the perfusion (*p* = 0.84) (Figure 15).

## Discussion

The pancreas is an organ vulnerable to edema and ischemic injury during retrieval and preservation leading to microcirculatory dysfunction [20]. This is likely one of the main reasons why perfusion of the pancreas did not gain as much interest as it has for other organs. In this study, all 6 cases were successfully perfused for the established time with a stable arterial pressure, flow, temperature, and a good macroscopic and microscopic appearance. Insulin increase was noted during the perfusion and glucose and lactate levels were close to normal by 4 h. This was similar to the results in our porcine model and these grafts were successfully transplanted with minimal evidence of injury, normal glucose tolerance tests, and no signs of pancreatitis [21].

The first NEVPP of discarded human pancreases was reported by Barlow et al in 2015 [24]. They reported 4 cases with a 2 h perfusion, proving technical feasibility but with poor results on histopathology and no mention of graft weight gain. According to this paper, there were 5 cases but, the last one had to be discarded because of an ischemic appearance during perfusion thought to be due to 30 h of CIT. For the fourth case, perfusion had to be terminated after 60 min due to low perfusate volume. All the cases showed a significant degree of necrosis, and the authors deemed this method to be feasible but not suitable in its current state.

In our study, grafts were perfused for a longer period with excellent tissue viability and close to normal morphological histopathology appearance after 4 h. Unlike Barlow et.al., our arterial perfusion pressure was set to 15–25 mmHg, instead of 50–55 mmHg [24]. A lower perfusion pressure was found to be critical for successful perfusion in the porcine model and appears to be similarly important in human grafts [21, 25]. Our system also incorporated a dialysis circuit which improved the degree of tissue edema that developed during perfusion. Finally, based on previous work, a protease inhibitor was added to the perfusate to mitigate any active enzyme that permeates into the system [21, 24].

According to studies in hypothermic machine perfusion of the pancreas, lower perfusion pressures obtain more stable perfusions and better results overall [19, 26]. Because of this, we decided to keep the pressure around 20–25 mmHg for the first 4 cases. In the latter part of the study, we noticed that when using a higher CO2 concentration, we could drop the pressure to 15 mmHg, without compromising the readings of pH, HCO3, pO2, or pCO2 concentrations in the perfusate. Cases 5 and 6 were perfused with a higher CO2 concentration (9% vs. 5%) which allowed a decrease in the overall arterial pressure.

The percentage change in wet/dry weight ratio before and after perfusion ranged from 6% to 42%. We noticed that the lowest change in ratio occurred in the grafts perfused with a higher CO2 concentration. Hypercapnia is a well-known vasodilator, but its use has been mainly described and studied for cerebral blood flow [27]. Studies in rats suggest that hypocapnia contributes to hypoperfusion and edema [28]. From our experiments in swine, we noted that increasing the CO2 concentration in the perfusate decreased the intravascular resistance which allowed us to perfuse the grafts with a lower arterial pressure, as mentioned previously. In this study, intravascular resistance was slightly lower but not significant in the CO2 5% group. However, only 2 cases were perfused in the CO2 9% group and more experiments are needed in this arm to confirm this trend. Edema has been a recurrent problem during perfusion [20, 25]. We believe that both the dialysis filter and higher CO2 contributed to small increase in water content, but further studies are needed.

Since this is a closed system, amylase increased during the perfusion, as expected, but this does not seem to correlate with damage to the graft, according to previous reports [29] and our histopathology results, which showed intact parenchyma (Figure 10). Lipase was measured during the experiments, however, the maximum range (3,000 U/L) was reached very early in the experiments and did not prove to be a useful marker. The development of better biomarkers to measure graft injury is needed and will enhance the ability of NEVPP to be used for graft assessment.

The most common complication after pancreas transplantation is vascular thrombosis, followed by graft pancreatitis. According to Nadalin et.al., a physiological acute pancreatitis occurs in 100% of the patients undergoing PTx, due to ischemia-reperfusion injury and this is typically clinically silent [30]. In our previous studies [21, 23, 31], with porcine models and machine perfusion we had no cases of vascular thrombosis or signs that physiological graft pancreatitis was not successfully resolving by the day of the sacrifice. None of the grafts in this study were transplanted after perfusion, but we could hypothesize that human grafts would do as well as the porcine grafts after transplant. This hypothesis could be supported by the histopathological findings that demonstrated minimal signs of apoptosis by TUNEL assay, minimal endothelial damage seen with CD31 staining and no apparent evidence of thrombosis. We hope that NEVPP when used with marginal grafts will help mitigate severe ischemia-reperfusion injury (IRI) which occurs post transplantation and eliminate/reduce IRI-related complications and allow for successful transplantation.

It has been established that reactive oxygen species (ROS) are an important injurious factor for ischemia reperfusion injury [32, 33]. The studies regarding levels of malondialdehyde (MDA) and machine perfusion are scarce and mainly refer to Hypothermic Machine Perfusion (HMP). In 2017, Kosieradzki et al studied 50 kidney transplant recipients. Grafts were procured from 27 brain death donors and preserved in a pulsatile perfusion device for a total mean ischemia time of 36.7 ± 8 h. They concluded that the 18 recipients that presented delayed graft function presented higher levels of MDA in the preservation solution at the end of the perfusion [34].

In our study, no significant difference was noted in MDA concentration in the perfusate at baseline and at the end of the perfusion, these results are in accordance to what was previously reported by Brüggenwirth et.al. in 2020 in porcine livers submitted to hypothermic and normothermic machine perfusion [35]. Interpretation of the results might prove to be difficult, but we could hypothesize that normothermic machine perfusion is useful to slow down or mitigate the oxidative stress.

Our study has several limitations. First, the complicated setup of our machine is difficult to replicate, and the cost of every experiment is high (around 5000CAD). The grafts were only perfused and not transplanted afterward, so no follow-up is possible. All the grafts were discarded so none of these grafts were from ideal donors and could have already presented some degree of damage before perfusion. Currently, there is no suitable test to assess the quality of the graft during perfusion, and we still do not fully understand the physiology of the pancreas during normothermic machine perfusion. The addition of a dialysis filter helped with the control of edema but makes the interpretation of glucose and lactate levels challenging as they will normalize over time. In addition, the number of grafts is limited, as mentioned previously, however, we believe the data demonstrates that normothermic machine perfusion in human pancreas allografts is feasible. Future studies will be directed towards better understanding the physiology while undergoing NEVPP.

In conclusion, normothermic machine perfusion of the human pancreas is feasible, maintaining both the macroscopic and microscopic appearance of the pancreas at the end of the perfusion and could prove to be useful for the assessment of extended criteria donors pancreases both for whole organ transplantation and islet isolation. Future studies will focus on the development of tests and biomarkers for the assessment of grafts. Identifying optimal perfusion settings and modifying mechanisms of inflammation could allow us to bring this novel technology to the clinical setting. Normothermic pancreas perfusion holds the promise to increase the pancreas donor pool by improving graft preservation, assessment, and repair.

## Data Availability

The raw data supporting the conclusion of this article will be made available by the authors, without undue reservation.
